# Supersensitive Worms Reveal New Gene Functions

**DOI:** 10.1371/journal.pbio.0000026

**Published:** 2003-10-13

The past ten years saw great progress in the field of molecular genetics, as new tools gave scientists the ability to investigate entire genomes instead of just one or two genes at a time. In this paper, Ronald Plasterk and colleagues developed a systemic approach using Caenorhabditis elegans, a tiny nematode and the first animal to have its genome sequenced, to gather functional information on nearly 400 genes.

Many of the systemic approaches to discovering gene function involve either measuring or deleting messenger RNA (mRNA), the molecule that helps translate genes into proteins. The method used here, called RNA interference, or RNAi, follows the deletion approach by taking advantage of a cellular process bearing the same name. In nature, RNAi is thought to be an important part of the innate defense machinery in plants and animals, protecting them from invaders like viruses by interrupting the manufacture of viral proteins. To do this, short double-stranded RNA molecules with complementary sequences to the target gene inhibit the gene's function by disabling mRNA, which effectively shuts down the gene. By mimicking this natural process to turn off selected genes, scientists can find clues to how those genes might normally function by watching what happens when they are taken out of the picture.

With the fully sequenced worm genome, it is possible to create interfering RNAs for all of its 20,000 or so genes. And because worms eat bacteria—which can themselves be used to deliver interfering RNAs—worms are the perfect RNAi model organism. The researchers fed the worms RNAi-producing bacteria, then observed the effects on the worm or its offspring to infer the function of the targeted gene. As previously reported, repeating this experiment for every gene in the worm genome, yields about 10% of the worms displaying abnormalities ranging from embryonic death to uncoordinated movement, suggesting defects in genes controlling development or muscle control, respectively.

Having previously identified an RNAi-hypersensitive mutant worm strain, Plasterk and his colleagues repeated the experiment in the mutants and report proposed functions for 393 previously unknown genes. The types of abnormalities observed in the short-lived mutations induced by RNAi, they say, resemble the more stable mutations seen in the collection of worm mutations cataloged by worm researchers over the years. Though the DNA alterations for many of these mutations are not yet known, researchers know roughly where they occur in the genome. And the researchers show here that they can use their RNAi experimental results along with what is known about the mutants to identify several of the sequence alterations. They also performed what is believed to be the first analysis in which independently generated large-scale RNAi results were systematically compared to see how variable such RNAi results are, and the results have implications for similar approaches not just in worms but in plants and other animals.

**Figure:**
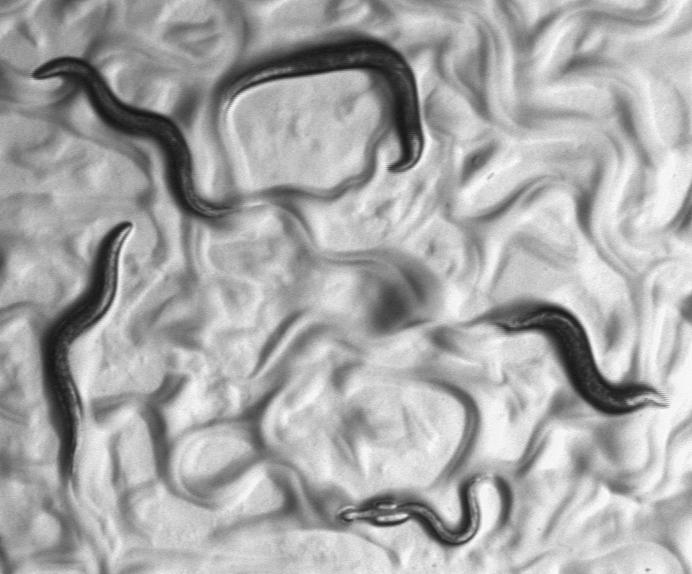
Caenorhabditis elegans worms

